# The effect of chiropractic treatment on the reaction and response times of special operation forces military personnel: study protocol for a randomized controlled trial

**DOI:** 10.1186/s13063-016-1580-1

**Published:** 2016-09-20

**Authors:** James W. DeVocht, Dean L. Smith, Cynthia R. Long, Lance Corber, Bridget Kane, Thomas M. Jones, Christine M. Goertz

**Affiliations:** 1 Palmer Center for Chiropractic Research, 741 Brady St, Davenport, IA 52803 USA; 2 Department of Kinesiology and Health, 26E Phillips Hall, Miami University, Oxford, OH 45056 USA; 3 Chiropractic Clinic, Blanchfield Army Community Hospital, 650 Joel Drive, Fort Campbell, KY 42223-5349 USA

**Keywords:** Chiropractic manipulative therapy, Reaction times, Response times, Special forces, Biomechanical assessments

## Abstract

**Background:**

Chiropractic care is commonly used to treat musculoskeletal conditions and has been endorsed by clinical practice guidelines as being evidence-based and cost-effective for the treatment of patients with low back pain. Gaps in the literature exist regarding the physiological outcomes of chiropractic treatment. Previous pilot work has indicated the possibility of improvements in response time following the application of chiropractic treatment. However, it is unknown whether or not chiropractic treatment is able to improve reaction and response times in specific populations of interest. One such population is the U.S. military special operation forces’ (SOF) personnel.

**Methods:**

This study is a randomized controlled trial of 120 asymptomatic volunteer SOF personnel. All participants are examined by a study doctor of chiropractic (DC) for eligibility prior to randomization. The participants are randomly allocated to either a treatment group receiving four treatments of chiropractic manipulative therapy (CMT) over 2 weeks or to a wait-list control group. The wait-list group does not receive any treatment but has assessments at the same time interval as the treatment group. The outcome measures are simple reaction times for dominant hand and dominant foot, choice reaction time with prompts calling for either hand or either foot, response time using Fitts’ law tasks for small movements involving eye-hand coordination, and brief whole body movements using the t-wall, a commercially available product. At the first visit, all five tests are completed so that participants can familiarize themselves with the equipment and protocol. Assessments at the second and the final visits are used for data analysis.

**Discussion:**

SOF personnel are highly motivated and extremely physically fit individuals whose occupation requires reaction times that are as quick as possible during the course of their assigned duties. A goal of CMT is to maximize the functionality and integration of the neuromusculoskeletal systems. Therefore, chiropractic treatment may be able to optimize the capacity of the numerous components of those systems, resulting in improved reaction time. The objective of this study is to test the hypothesis that CMT improves reaction and response times in asymptomatic SOF personnel.

**Trial registration:**

ClinicalTrials.gov, NCT02168153. Registered on 12 June 2014.

## Background

Chiropractic manipulative therapy (CMT) is generally used to treat musculoskeletal conditions, with a focus on spinal health. Spinal manipulation (SM) is the primary chiropractic intervention [1]. Multiple clinical practice guidelines have endorsed CMT as being evidence-based and cost-effective for the treatment of patients with acute, subacute, and chronic low back pain (LBP) [2–4]. These guidelines are based upon randomized controlled trials (RCTs) that demonstrate SM to be a conservative and effective approach for the treatment of LBP [5–8]. In the U.S., between 7 and 14 % of U.S. adults see a doctor of chiropractic (DC) annually, resulting in more than 190 million patient visits and there are more than 70,000 licensed DCs [9–12]. CMT receives high patient approval ratings in studies done to assess patient satisfaction [13–16]. In addition to private practice, DCs treat patients in a variety of settings including multidisciplinary health care organizations such as Veterans Health Affairs and military treatment facilities [17, 18]. Currently, DCs provide treatment in 65 military treatment facilities both within and outside the U.S. [19].

SM is used by professional sports teams to enhance player performance. Currently, there is some preliminary evidence that CMT may have a positive effect on both reaction time and movement time [20, 21]. Kelly et al. found that participants demonstrated a significant improvement in a complex reaction time task after receiving CMT [22]. Both Smith et al. [23] and Passmore et al. [24] reported that hand and head movements in response to visual stimuli were completed more quickly after participants had received CMT. Daligadu et al. reported that 10 volunteers with subclinical neck pain were able to complete specified sequences of button presses on a keypad more quickly after receiving CMT [25]. No adverse events (AEs) were reported in any of these studies.

One group that relies heavily on peak physical performance is special operation forces (SOF) of the U.S. military. Enhanced performance is critical for this population as they encounter dangerous situations. Split-second delays in response times to threats may mean the difference between life and death. It is for this reason that the Office of the Congressionally Directed Medical Research Programs issued a Program Announcement that led to the Defense Health Program Chiropractic Clinical Trial Award (W81XWH-11-2-0107) to, in part, “assess military readiness by evaluating pre-post differences in reflexes and reaction times following chiropractic treatment using a pre-post interventional cohort trial in members of Special Operation Forces.” In response, the objective of this study is to test the hypothesis that CMT improves the reaction and response times of these highly motivated and extremely physically fit individuals.

## Methods

### Overview

**Fig. 1:**
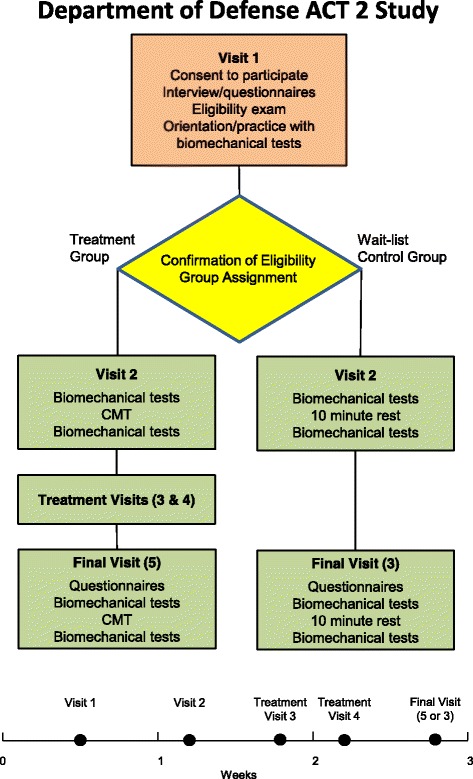
Assessment of Chiropractic Treatment, part 2 (ACT 2) study flow chart and timeline

This study is a RCT measuring reaction and response times in 120 volunteer SOF personnel at the Blanchfield Army Community Hospital, Fort Campbell, KY, USA. Following a first visit for screening and practicing the five biomechanical tests to be used in assessments, participants are randomly allocated to either a treatment group or to a wait-list control group. Beginning within a week of their first visit, those in the treatment group receive four CMTs over 2 weeks. The first of two assessments consisting of five biomechanical tests is made during their second visit, along with their first CMT. The second assessment is made during their final visit, along with their fourth CMT. In both of those visits with assessments, some of the biomechanical testing is performed before the CMT and some performed after. Participants in the wait-list control group do not receive any treatment but complete the two biomechanical assessments at the same time intervals as those in the treatment group. A flow chart of the study is shown in Fig. 1. Following their involvement in the study, those in the wait-list control group are offered the opportunity to receive four CMTs.

### Trial organization

This RCT is being conducted at Fort Campbell, KY because it has a population of SOF personnel as well as an established chiropractic clinic. The space to conduct the study, including the equipment used in the biomechanical tests, is housed within the facility used by the Chiropractic Clinic at Blanchfield Army Community Hospital. The CMT for this study is provided by two DCs, each with more than 10 years of clinical experience, practicing under the auspices of clinical guidelines established by the Department of Defense (DoD) and MedCom.

The investigators forming the research team for this study are from three collaborating institutions: the RAND Corporation, Palmer Center for Chiropractic Research (PCCR), and the Samueli Institute. Grants administration is managed by the RAND Corporation including the financial aspects and Institutional Review Board (IRB) issues of the grant award. It also ensures that the program officer at the DoD receives the required deliverables. The Samueli Institute ensures that the study complies with those entities that regulate the conduct of human subjects’ clinical research within the DoD, which include the U.S. Army Medical Research and Material Command Human Research Protection Office and the Army’s Clinical Investigation Regulatory Office. The Samueli Institute also provides advice concerning the general processes associated with the conduct of research within the military community.

Investigators from the PCCR are responsible for developing, implementing and managing the RCT at Fort Campbell. The investigators at Fort Campbell include: the site project manager (PM), two DCs (one of whom serves as site project investigator (PI)), and a physician medical monitor. The site PM is responsible for day-to-day trial implementation including recruitment and enrollment of trial participants, administration of a practice session and two biomechanical assessments of each participant, ensuring that each participant completes all phases of the study within the prescribed time windows, recording AEs, and maintaining all site-level trial documentation. The site PI oversees site administration including IRB issues, monitors study progress, conducts study evaluation and CMTs and ensures that all study procedures are conducted according to the protocol.

The lead PM at the PCCR oversees trial operations at Fort Campbell, acts as a liaison between trial coinvestigators, and ensures protocol adherence and fidelity. AEs are reviewed and monitored by a clinician at the PCCR. The project committee, consisting of all PCCR personnel involved in the study, meets weekly to review progress and resolve any issues that may arise. Any potential changes to the protocol are discussed in these meetings. Action steps are determined for obtaining approval from the three IRBs and informing all relevant study personnel.

The Submission Tracking and Reporting System (STaRS) used in this RCT is a comprehensive web application developed by the PCCR with a dual purpose of collecting outcome assessments for study participants and serving as a secure electronic data capture and clinical trial management system. STaRS includes modules for confirmation of participant eligibility, biomechanical assessment file exchange, data collection of study participant’s outcome assessments, and real-time reports for study management.

### Data and Safety Monitoring Committee

A Data and Safety Monitoring Committee (DSMC) provides oversight for this study. All DSMC members are independent of Palmer College of Chiropractic. Responsibilities of the DSMC are: (1) to ensure the overall safety of participants in clinical trials conducted by PCCR investigators by protecting participants from avoidable harm, and (2) to advise the DoD and the Expert Advisory Board regarding the scientific and ethical conduct of this RCT.

The DSMC reviews reports biannually. Should an AE occur, the DSMC evaluates the related data to protect the safety of study participants. If necessary, DSMC members make recommendations to the PIs and the DoD regarding continuation, termination, or other modifications of the RCT.

### Recruitment procedures

#### Initial contact

Flyers describing the study are placed in SOF facilities at Fort Campbell. SOF unit commanders and health care providers assigned to deliver care to SOF help to identify appropriate methods for dissemination of information concerning this study to their personnel. Quarterly presentations about the study are made in the language school on post as each new class begins. SOF soldiers who are interested contact the site PM by phone or email. The PM briefly describes the nature and extent of the study and asks basic screening questions. If the potential participant is still interested and appears to be eligible, the site PM arranges a preliminary visit to the study location for more extensive screening in a private setting.

### Visit 1

At the first visit, the site PM explains the study in detail utilizing the study flow chart and describing the specific activities of each visit. The site PM then goes over the Informed Consent Document with each participant and gives them a chance to read it and a Health Insurance Portability and Accountability Act (HIPAA) Compliance Document. The site PM is available to answer any questions they may have about either document or any aspect of the study. If the individual still desires to enroll in the study, the participant signs both documents and the site PM signs as a witness. The site PM then conducts an interview in which basic demographic information is obtained. The PM also screens the participant based on nonclinically obtained eligibility criteria. Those criteria are shown in Table 1 along with a rationale for why each was included. The PM enters the participant information directly into the STaRS system. Once preliminary eligibility is determined, the participant logs into the secure web application participant database (STaRS) and completes a demographic information form, a health care utilization and medication use form, and the Patient Reported Outcomes Measurement Information System (PROMIS)-29 Health Survey. After the questionnaires are completed, one of the two DCs in the chiropractic clinic reviews the participant’s medical records and conducts a physical examination. If no contraindications to chiropractic care are identified by the DC, the participant is referred back to the PM to complete the remainder of the visit. Participants with identified contraindications to CMT are ineligible to participate and referred to an appropriate provider if other care is needed. Those who are eligible are given an orientation of the five biomechanical tests. The orientation includes three videos, each approximately 2 or 3 min long, which demonstrate and narrate each of the five different tests..They show each test being performed and explain how the timing is measured for each one. Then the participant practices the five different biomechanical tests, repeating each one three times. The instructional videos were created to ensure that each study participant would receive standardized instructions. An appointment is then made for visit 2 within a week of visit 1.

**Table 1:** Eligibility criteria

Inclusion criteria	Rationale
Minimum age of 20 years	Minimum age of SOF personnel
Written informed consent	Must be able to understand and agree to the requirements of the study
Active duty special operation forces’ (SOF) personnel stationed at the Fort Campbell, KY military site	SOF personnel are the focus of this study. Fort Campbell is the study site
Exclusion criteria	
Pain intensity ≥4 (using the National Institutes of Health’s PROMIS – question #29) at the initial visit	High pain levels have the potential to confound study results
Additional diagnostic procedure (other than X-ray) or referral required to determine a diagnosis, obtain a second opinion, or to manage a condition	Additional clinical diagnostic procedures are beyond the scope of this study
Bone and joint pathology contraindications for chiropractic manipulative therapy (CMT). Potential participants with conditions such as recent spinal fracture, concurrent spinal or paraspinal tumor(s), spinal or paraspinal infection(s), inflammatory arthropathies and significant osteoporosis	Participant safety. Care outside study scope needed
Other contraindications for CMT or suspicion of such contraindication requiring a consultation with another provider (i.e., unstable spinal segments, suspected cauda equina syndrome)	Participant safety. Care outside study scope needed
Currently being treated for traumatic brain injury	Potential to confound study results
No known or pending deployment, orders for a distant duty assignment or training site, or other absence from the current military site during the study participation period (2–4 weeks)	Compromises ability to adhere to study protocol
Received care from a doctor of chiropractic within the past 30 days	Prevent possibility of carryover effects from recent chiropractic care

There have been two changes in the eligibility criteria since this study began. Originally, the upper age limit was set at 45 years. However, as recruitment progressed it became apparent that SOF included members over the original age limit with an interest in the study. Consequently, it was decided to allow those who were still active in SOF to participate with no upper age limit. The second change allowed women to participate in the study. Initial study recruitment was limited to personnel who were not only in SOF, but were also special forces-qualified – a subset of SOF personnel who could not be female. In order to meet the required rate of recruitment, it was necessary to broaden the scope of eligibility to include members of the Special Operations Aviation Regiment who are on flight status, which includes female pilots. Both changes were approved by all IRBs involved in this study. The changes were made after 41 participants had been enrolled in the study.

### Between visit 1 and visit 2

The data from visit 1 is entered into STaRS. If all eligibility criteria are met, the participant is randomly allocated to either the treatment group or the wait-list control group. Group assignment is done using concealed allocation in a 1:1 ratio by a predetermined, computer-generated, restricted randomization scheme with random block sizes of 2, 4 or 6. The site PM has no knowledge of any details of the randomization process but accesses the group allocation module within STaRS to retrieve the participant ID and assigned group. The group assignment and date, time, and study participant ID are stored in the Structured Query Language (SQL) database. If the STaRS database is unable to be accessed when a participant needs to be allocated to a group, there is a backup allocation protocol consisting of predetermined sequentially numbered, opaque envelopes. Once allocation has been made to either the treatment group or the wait-list control group, the participant is called or sent a text message specifying their group assignment. Personnel at the PCCR who process the raw data are blinded to which group individual participants have been allocated to and will remain blinded until after completion of the study.

### Study interventions

The criteria for determining the clinical appropriateness for CMT are similar for the minimally symptomatic (current pain intensity no more than 4/10) and the totally asymptomatic participants of this study. The DCs perform a clinical evaluation, which may include standard orthopedic tests, spinal ranges-of-motion assessments, gross movement patterns, paraspinal muscular evaluation, and spine-related palpatory examinations to identify areas that may respond to CMT. Clinicians may use findings such as point tenderness over the spine, local muscular hypertonicity, asymmetry in posture, or pain/tenderness produced with orthopedic examination maneuvers to provide information regarding the appropriateness of spinal manipulation. In this manner, clinical evaluation can reveal musculoskeletal dysfunction in otherwise asymptomatic patients.

When applicable, the DCs decide which specific form of CMT to use based primarily upon the diagnosis and combination of comorbid or complicating diagnoses, if any. The participant’s previous response to care (if known), flexibility and mobility, and general condition are also considered. The study chiropractor then makes a second decision regarding the application (location and direction) of CMT to the spine. This decision is based upon the diagnosis and other examination findings such as tenderness, hypertonicity, hypomobility, positions of relief and provocation, imaging findings (e.g., spinal curvatures, degeneration, spondylolisthesis) and other factors individual to the case. The care given to any individual participant consists of high-velocity low-amplitude (HVLA) spinal manipulative procedures. These procedures are typically associated with a quick manual thrust and an accompanying cavitation sound. For the cervical spine, the DCs use a cervical diversified technique. Thoracic manipulation occurs with unilateral or bimanual contacts in the prone or supine positions. Lumbar/pelvis manipulation is performed with a procedure referred to as side-lying or side-posture.

### Visit 2

At visit 2, the site PM shows the same instructional videos that were seen at visit 1 to the participant just before the participant performs the biomechanical tests. The participants are first asked to complete two repetitions of each of the five biomechanical tests. Following the two repetitions of the five tests, those in the treatment group receive their first CMT. Those in the wait-list control group wait for 10 min, the typical amount of time for a CMT to be given. Participants of both groups then complete one more repetition of each of the five biomechanical tests.

### Visits 3 and 4

Participants in the treatment group come in for two more visits and receive CMT over the next week with no biomechanical assessments. Participants in the wait-list control group do not attend these visits.

### Final visit

The final visit of the study is the fifth visit for those in the treatment group and the third visit for those in the wait-list control group. At the beginning of this visit, the participant logs into STaRS and completes the health care utilization and medication-use form and the PROMIS-29 questionnaire, as was also done during visit 1. The five biomechanical tests and CMT/break are conducted in the same manner as at visit 2, which marks the completion of an individual’s participation in the study. Those who are in the wait-list control group are then offered the opportunity to receive CMT. If desired by the participant, the first of four CMTs is given at the final visit of their participation in the study after completion of the biomechanical tests.

### Missed visits

Due to the nature of SOF missions, unexpected deployments or local mission essential requirements can occur. Consequently, there are times when participants are not able to complete the study visits within the normal 2-week time window. However, participants must complete each of the visits for the study in the prescribed sequence. In the event of a missed appointment, the site PM contacts the participant to reschedule. It the study visits cannot be completed within 4 weeks, the PI and site PM discuss additional scheduling options on a case-by-case basis. If all visits are unable to be completed, the participant is considered as lost-to-follow-up.

### Outcome measures

Reaction times are typically very quick (less than 1 s). Subsequent changes in reaction time would be shorter still. Consequently, any tests to be used in this study must be very precise. Reaction time is the time from when a prompt is presented to the beginning of movement in response to that prompt. Response time is the time from when a prompt is given to the completion of a specified task. Three outcome measures are used that involve only a slight degree of movement, so the response time is essentially the same as the reaction time. Two additional outcome measures involve a higher degree of motion and require a longer period of time from the prompt to the response completion. Therefore, the length of time required to complete those tasks is more accurately referred to as response time. However, the movement required for these two outcome measures is still quite small – the response time for each event is still usually less than 1 s.

Before data collection was initiated at Fort Campbell, a pilot study was conducted at Palmer College of Chiropractic to develop and refine the specific procedures for each outcome measure. The three reaction time tests and two response time tests used in this study are described below. Due to the lack of information in the literature concerning these five biomechanical tests, no specific one of the five tests was selected as a primary outcome measure.

#### Simple reaction time of the dominant hand

Handedness of the participants is determined on the basis of self-report. The participant sits in front of a computer screen holding a button in their dominant hand and reacts to the appearance of visual prompts on the screen by pressing the button. A series of 11 prompts are shown in sequence. The time period between the response to one prompt and the appearance of the next prompt ranges from 0.5 to 4 s in a random although set sequence. The outcome variable for this test, the mean reaction time, is the average length of time between the appearance of each of the last 10 prompts and the button pressed in response to that prompt.

#### Simple reaction time of the dominant foot

This test is the same as the test with the dominant hand (previous paragraph), except that the participant’s response to the visual prompt is made by pressing a pedal with the dominant foot.

#### Choice reaction time

This is a reaction time test involving both hands and both feet. The participant sits in front of a computer screen with a button in each hand and each foot resting on a pedal. A set of 41 prompts appear sequentially on the screen. The position of the prompt on the computer screen, as well as text within the prompt, indicates which hand or foot should be used in response to each prompt. If the prompt is in the upper left corner, the subject presses the button with their left thumb. If the prompt is in the upper right corner, the subject presses the button with their right thumb. If the prompt is in the lower left corner, the subject presses the left foot pedal, and if the prompt is in the lower right corner, the subject presses the right foot pedal. There is a 1-s interval between the press of a button or pedal in response to a prompt and the appearance of the next prompt. If the wrong button or pedal is pressed, the software still goes on to the next prompt, but keeps track of how many incorrect responses were made and which ones were incorrect. The outcome variable for this test, the mean reaction time, is the average length of time between the appearance of each of the last 40 prompts and the press of a button or pedal in response to that prompt. However, the reaction times corresponding to incorrect choices are not included in the mean, in accordance with the protocol described by Whelan [26]. The number of incorrect choices is also provided.

#### Response time involving the dominant hand (the Fitts’ law test)

In this test, participants perform a computerized, simple target-acquisition task (known as a Fitts’ law task) to investigate their response times using a mouse with their dominant hand. The participant completes a block, a series of target selections on a computer screen, by working through 32 trials. That is, 32 pairs of “hits” – meaning the mouse is clicked when the cursor is inside each of two circles that make up a pair. When a pair is completed, the screen goes blank. The participant can then click the mouse with the cursor anywhere on the blank screen to begin the next of the 32 pairs in that block. This process continues until all 32 pairs of that block have been completed.

**Fig. 2:**
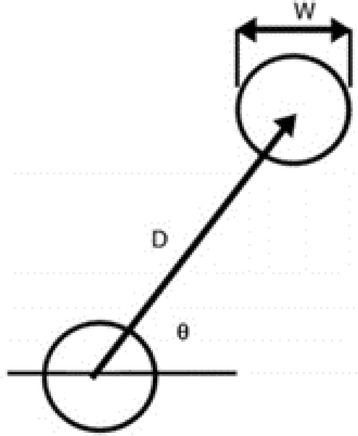
Computer screen used with the Fitts’ law test. The participant moves the cursor from one circle to the other and back. The process is repeated for 32 pairs of circles of different size and orientation

The two circles of any given pair are always of equal size, although the size varies in a random but set manner from pair to pair (W in Fig. 2) as does the orientation of the circles on the screen (the angle θ in Fig. 2). The distance between the centers of the two circles (D in Fig. 2) is always the same for every pair.

The participant is given a practice block of five trials before completing this task in order to become familiar with the process involved. The measured outcome from this task is the sum of the times required to complete each of trials (pairs) in a 32-trial block. The time elapsed between pairs is not counted.

#### Response time involving whole body movement (t-wall)

**Fig. 3:**
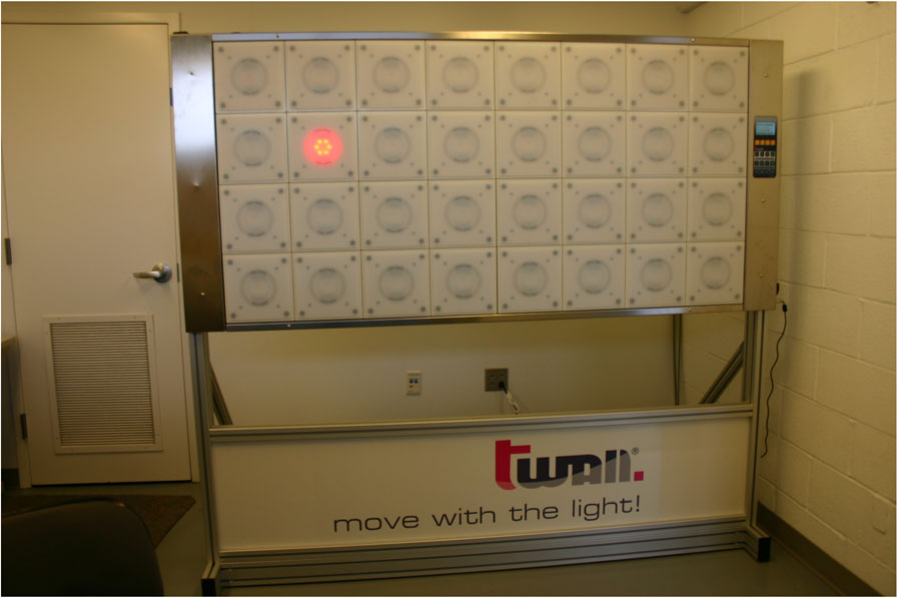
The t-wall. The participant goes through a random sequence of striking 100 lighted buttons, one immediately after the other

Participants stand in front of the t-wall, a commercially available device (Motion Fitness, Rolling Meadows, IL, USA) with a 4 × 8 bank of square buttons each of which is 8 cm per side (Fig. 3). When the test begins, one of the buttons will light. The participant hits that button with either hand. The light inside that button then goes out and another button lights until hit. This process continues for a random sequence of 100 buttons. When the last button is hit, all the buttons flash once to indicate that the test is complete. Participants are given a practice run on the t-wall to familiarize them with the process and the amount of force required in order to constitute a hit on any of the buttons. The starting position is standing an arm’s length way from the center of the device. Initially, the first button of the 100 sequence is lit. However, the timing does not begin until the participant hits that first button. The measured outcome from this test is the time from when the first button is hit to when the last button in the random but set sequence of 100 buttons is hit.

#### Sequence of biomechanical testing

The tests for each of the five reaction and response time outcome measures are given three times on each of the three different visits. Two different random but set sequences of prompts, designated A and B, are used for each of the five tests. This is done to prevent the participants from memorizing the sequence of prompts and, therefore, being able to anticipate the next prompt.

Although the five tests are all given at visit 1, it is only for practice – the data is not used in the analysis of the study. This allows the participants to become familiar with each test in preparation for the two assessment visits. The sequences of prompts as used at visit 1 are alternated for the three repetitions of each of the five tests using the pattern of A, B, A.

During visit 1, a video explaining the use of the t-wall is shown to the participant. The participant is then given the chance to hit a few buttons before actually beginning the test. This allows the participant to experience how much force is required when hitting a button in order to make the light go out. Once comfortable with this concept, the participant goes through three repetitions of the 100-button test. Next, the participant is shown a second video about performing the Fitts’ law test, followed by a sample Fitts’ law test of five pairs of circles. Once the participant feels able to do the test smoothly, three repetitions of that test are completed with 32 pairs of circles in each repetition of the test. After completion of the Fitts’ law tests, a third video is shown that explains and demonstrates the hand and foot simple reaction time tests and the choice reaction time tests. The participant then performs three repetitions of each of those three tests. This denotes the completion of visit 1.

The first assessment is done during visit 2 and the second assessment is done 10 days later during the final visit. For each assessment, the five different biomechanical tests are given in the same sequence as they were practiced at visit 1. This includes showing the instructional videos before the relevant type of test but does not include doing the brief sample before performing the t-wall and Fitts’ law tests. First, two repetitions of each test are performed. Then, the participant receives either a CMT or, for those in the wait-list control group, a 10-min break. After the CMT (or break), a third repetition of each type test is given – with the five tests being given in the same order. For these two assessment visits, the two sequences of prompts for each of the five tests and the CMT are given in the order of B, A, CMT/break, A. The videos are not shown again for the tests that are completed after the CMT or break.

### Software for the computer-based tests

The programs that are used for the computer-based tests were custom-developed using the Paradigm software package (Perception Research Systems, Inc.). The Fitts’ law test uses the computer mouse for the participant to interact with the program. The reaction and response time tests use hand-held buttons and foot pedals for the participant interaction. The MP150 Data Acquisition System (BIOPAC Systems, Inc.) is used to interface the output of the buttons and pedals with the reaction time testing programs that were developed with Paradigm software.

### Data collection

Patient demographics are collected at visit 1. Health care and medication use and the PROMIS-29 are administered during both visit 1 and the final visit. A checklist is used that contains a list of each repetition of each of the five biomechanical tests in the order that they are to be given. Each test is checked off as it is completed.

Four of the five biomechanical tests that are administered to each participant are performed with the participant interacting with a computer: simple reaction time test with the hand, simple reaction time test with the foot, choice reaction time, and the Fitts’ law test. A data file, in the form of a Microsoft Excel spreadsheet, is generated by the computer each time that one of those four tests is given. Since each of those four tests is given three times during each visit, there are 12 Excel files generated for each participant (visit 1 – just for practice, visit 2, and final visit). The name of each Excel data file contains the ID of the participant, a letter indicating when that data was taken: visit 1 – for practice only, visit 2, or the final visit. Also included is the date that the data was collected in the format of yy-mm-dd, as well as a letter and a number to indicate which of the five biomechanical tests, and which repetition of that test, generated the data.

The result from each repetition of the test using the t-wall is a single number representing the time in seconds (to two decimal places) that it took a participant to complete pressing the 100 buttons that constitute a repetition of that test. That number is shown in a digital display on the t-wall device. Immediately after a repetition of the t-wall test is completed, that number is written on the checklist. Consequently, there are three numbers for the t-wall that are hand-written on the hard copy checklist during the course of each visit.

### Data management and security

There are 12 Excel files that are generated during each visit in which data are collected. Those files are later combined into a single zip file. Consequently, there are three zip files created for each participant - each one containing the 12 Excel files from one of the visits. Once a zip file is created following a visit, it is uploaded into the STaRS system.

At the PCCR, the zip file is downloaded from STaRS and stored on a secure file server on the PCCR network. All PCCR servers reside behind a state-of-the-art firewall with permissions determined by Active Directory. Through the use of a custom-developed macro in Excel, the individual data points are taken from each Excel data file and copied into a single large Excel file that acts as a database containing all of the data from all of the Excel data files. There is a separate sheet in the database file for the data of each type of test that generates an Excel data file: simple hand reaction time, simple foot reaction time, choice reaction time, and Fitts’ law data. In addition, there is a fifth sheet that also contains the choice reaction time data, except that in that sheet there are blanks instead of data for those choices that were incorrect. For example, in the case when the test presented a prompt that called for a response with the left hand, but the participant used the left foot instead of the left hand. Generating a sheet of the choice reaction time data in this manner provides the opportunity for that data to be analyzed excluding times from incorrect choices, as described by Whelan [26].

There is one line created in the database file for each repetition of each type test with all of the data from that test. That line also contains the participant ID, visit number (1, 2, or 3), the type of test, which repetition of the test for that visit (1, 2, or 3), the date the test was given, and the name of the data file from which this particular set of data originated. In addition, the sheets for the choice reaction time test also include the number of incorrect choices that were made on that instance of the test.

A custom macro for Excel was developed to permit t-wall data for a given visit that had been hand-written on the hard copy checklist to be key-entered into an electronic form that has a field for each bit of data. The data thus entered consists of the participant ID, visit number, repetition number of the t-wall test on that visit, and the three times that were taken to complete the three repetitions of the t-wall test in that visit. The macro places the values on the electronic form into the correct places in an Excel file that serves as a database for all the t-wall data. In that database file there is one line for each participant. That line contains all of the t-wall data for that participant along with the participant ID and the date of each visit in which data was collected. Quarterly onsite audits are made by the PCCR project manager to ensure that the times of all repetitions of the t-wall tests, as manually written on the data collection forms, have been accurately entered into the computer.

Excel database files are backed up monthly and placed on two other hard drives. The data core manager writes programs in the SAS System for Windows (Release 9.4; SAS Institute Inc., Cary, NC, USA) using SAS ACCESS to create the analyzable datasets and creates the data dictionary. Only the data core manager and biostatisticians will have access to the datasets.

### Statistical methods

An intention-to-treat approach, in which participants will be analyzed according to their original treatment allocation, will be used. All observed data will be used in the analyses. Data analyses will be performed using SAS. The level of significance will be set at 0.05. Descriptive statistics of participant baseline characteristics, the reaction and response times and the PROMIS-29 scales at visit 1 will be presented for each treatment group.

The primary analyses compare the mean changes in reaction and response times from sequence A, performed before CMT/break at visit 2, to sequence A performed before CMT/break at the final visit between the treatment and wait-list control groups, using an analysis of covariance controlling for age, for each of the five biomechanical tests. Residual plots will be used to assess the validity of the model assumptions. If group variances are heterogeneous, we will use a mixed-effects regression model. If the data is non-normal, we will explore data transformations. Mean differences between groups, adjusted for age, will be reported with 95 % confidence intervals.

The secondary analyses will compare the immediate changes in sequence A before CMT/break to sequence A after CMT/break at both visit 2 and the final visit using the same methods described above.

Although we do not expect changes in the PROMIS-29 Health Survey scales or medication use in this short time frame, we will explore it by analyzing changes from visit 1 to the final visit.

### Sample size

A power analysis used the standard deviations of mean changes in response/reaction time over a 1-week period for each of the five biomechanical variables obtained in the pilot study. We estimated effect size as a 10 % change of the mean response/reaction time measured at visit 1 for each variable, assuming the control group would have no change. A total sample size of 100 participants, with 50 per group, gives at least 85 % power to detect a 10 % difference in mean change between groups at a 0.05 level of significance. We increased the sample size to 120, with 60 per group, to account for the possibility of up to 15 % loss-to-follow-up.

### Internal quality assurance process

The lead PM conducts an internal quality assurance audit on a quarterly basis for the purpose of maintaining data integrity, ensuring study protocol fidelity and sustaining study operating procedures. During the audit, the lead PM reviews regulatory documentation and Informed Consent Documents. Electronic data is verified by comparing the paper source documents to the data entered into STaRS. Any errors discovered during the quarterly audits are documented, corrected by the site PM, and reported to the site PI, collaborating investigators, and appropriate regulatory bodies if applicable. During these site visits, the lead PM also meets with the site PM, PIs, DCs, and/or clinic command to facilitate communication about overall study status and discuss study timelines, as well as address site concerns or barriers interfering with study conduct. Information gathered during the site visits is conveyed to study coinvestigators. In addition, the PCCR PI has a monthly conference call with the lead PM and onsite PI and PM to monitor study progress.

### Adverse events

For this study, an adverse event (AE) is defined as any untoward medical occurrence that may present itself during the conduct of the study and that may or may not have a causal relationship with the study procedures. AEs are monitored at two levels: (1) a participant self-report AE collected at all visits, and (2) serious adverse events (SAE) regardless of their attribution. Both are reported directly to the site PM, the site PI, and the medical monitor.

There is few rigorously collected data available reporting the risk of AEs following CMT. The lack of quantifiable information is in part the result of the inherent challenges presented by defining and identifying AEs in patients with musculoskeletal complaints with natural symptom variation, the large number of modifiable procedures available to DCs, and the combination of adaptable procedures in varying patient populations [27].

The scientific literature does contain case reports of SAEs such as fractures, serious neurological symptoms, and cauda equina syndrome following CMT. However, case reports are anecdotal in nature and lack definitive causal links. In addition, there are very few case reports of SAEs relative to the total number of chiropractic visits. Therefore, the risk for SAEs following chiropractic care is extremely small and implausible to estimate accurately [28]. The most recent systematic review on this subject failed to identify any reported SAEs resulting from chiropractic care in clinical trials [28].

AEs and the anticipated likelihood of each for this study are included below:

- Rare but serious (event rate <1 %)
  - Fracture to the ribs or hip
  - Nerve injury that may cause loss of bowel or bladder function, lower body sensation or leg paralysis
  - Strokes
- Less likely (1 % ≤ event rate < 5 %)
  - Inadvertent disclosure of data
- Likely (5 % ≤ event rate < 10 %)
  - Some individuals may also experience: neck pain; headache; radicular (radiating) pain; mid-back pain; hands or feet tingling, burning, pricking, or numbness; or dizziness following neck manipulation. These symptoms are usually self-limiting and short-lasting
- More likely (event rate ≥10 %)
  - Some participants may experience muscle and/or joint soreness associated with palpation and CMT, particularly at the beginning of the program

Oversight of the reported AEs is conducted by a designated study clinician who reviews a dynamic report of all information submitted by the site PM using the secure web module designed for event reporting for this study. The designated study clinician conveys classification of these events to the site PM for appropriate reporting to the IRBs and other required regulatory bodies. The study clinician may also ask the site PM to contact the participant if more information is needed regarding a reported adverse experience that is potentially serious, related to the study, appears to have no resolution date, or appears to require additional medical follow-up for safety purposes. Our goal is to ensure that we are following up any event that has the potential to affect participant safety and reporting AEs per all study IRB reporting guidelines.

For the second level of AE monitoring, we use the Food and Drug Administration (FDA) definition of a SAE. This is any adverse experience occurring during treatment that results in any of the following outcomes: death, a life-threatening adverse experience, inpatient hospitalization or prolongation of existing hospitalization, a persistent or significant disability/incapacity, or a congenital anomaly/birth defect.

Should any arise, all SAEs and unanticipated problems involving risk to subjects or others are reported to the involved IRBs (Dwight D. Eisenhower Army Medical Center, Palmer Center for Chiropractic Research, and the RAND Corporation), Medical Monitor, DSMC, and the U.S. Army Medical Research and Material Command Office of Research Protections according to the relative reporting guidelines for each entity. The site PM is responsible for reporting all AEs in STaRS and to the lead PM at the PCCR. The lead PM at the PCCR is responsible for ensuring all appropriate parties are informed about any SAEs.

All study protocol violations are reported to the Palmer DSMC. Protocol violations meeting study site’s IRB criteria for reporting are reported per IRB guidelines.

### Study limitations

One limitation of this study is the use of a wait-list control group for comparison to the treatment group rather than a sham treatment group. A sham treatment would maximize the ability to compensate for the possibility of a placebo effect in the treatment group. However, it is very difficult to provide a sham treatment for HVLA active treatment, so the members of a sham group would likely suspect that they are not receiving an active treatment [29]. Furthermore, the outcome measures used in this study are objective in nature, potentially minimizing any placebo effect.

A limitation inherent in any study involving manual therapies is the variability of the treatment provided for each patient, and even each treatment of each patient. There are so many variables associated with virtually any form of manual therapy that it is impractical to try to quantify them. This is partly due to the fact that each patient’s condition is certainly not constant from one visit to the next, and consequently the manual therapy given is typically modified by the treating clinician to address the specific needs of the patient during any particular visit. This study is intended to investigate the effects of actual clinical practice and, therefore, no attempt was made to restrict the manner in which the clinicians provide their treatment to participants of the study.

Similarly, despite the requirement for participants to meet the inclusion criteria established for the study, there will still be considerable variations within the exact physical condition, some of which are likely associated with age, of the participants in the study. This is also an inherent limitation in studies involving human participants.

Another limitation of the study involves blinding. Due to the setup for this study, it is not logistically feasible for the person administering the assessment tests to be blinded as to which group (treatment or wait-list control) each participant is in. Consequently, there is a possibility that the assessor’s actions toward members of the treatment group might be somewhat more positive or encouraging than their actions toward members of the wait-list control group. This risk is minimized by having scripted dialog and prerecorded video presentations to explain how each assessment test is to be done. The objective nature of all five of the assessment tests reduces the ability for observer bias to impact the results of the tests.

One other limitation of the study is the low number of CMTs being given to each participant. It is currently unknown if CMTs of any quantity would induce a discernable reduction in the reaction time of SOF personnel. Three or four CMTs were chosen as feasible for busy SOF personnel to receive.

## Discussion

SOF personnel as a group are likely to be in need of reaction and response times that are as quick as possible during the course of their assigned duties. One goal of CMT is to maximize the integration and function of the neuromusculoskeletal systems. Therefore, this intervention is well-suited for attempting to optimize the capacity of the numerous components involved in the production of a minimal reaction and response time. This study is designed to show if CMT will result in quicker reaction and response times for those SOF personnel who receive it. The results of the study will be published following completion of data collection and analysis.

Two different random but set sequences of prompts for each of the five biomechanical tests were used to prevent participants from memorizing the sequence and anticipating prompts. However, different sequences can have different levels of difficulty. Consequently, whenever the results of two replications of the same test are to be compared with each other we wanted to have the same sequence of prompts used for those replications of that test. The only replications of each test that are to be used in the data analysis of this study are the ones taken immediately before and after the participant receives a CMT/break. Therefore, for visit 2 and the final visit, the order of prompt sequences and the CMT/break that is used is B, A, CMT/break, A. Having a replication of the test completed using sequence B just before the one with data that will be analyzed that uses sequence A has a double value. Not only does it prevent memorization of the prompt sequence, but it also provides an opportunity for the participant to get in the mode of doing that particular test after doing other, different tests before doing it for analysis.

Once the resulting manuscripts have been published, datasets will be provided for public access. Potential investigators can contact one of the co-PIs to present their hypothesis, study design, instruments and/or data on which to focus, and resources required. Depending upon the needs and desires of the requesting party, the data that is shared may include analytic tables or deidentified or limited datasets that are transmitted to the requesting parties for additional analyses. In addition, the trial is registered on ClinicalTrials.gov, making all key information about the trial freely available.

### Trial status

The first participant was enrolled on 30 September 2014. Data collection for the last of 120 participants was completed on 7 June 2016.

### Trial registration

The RCT discussed in this article was registered on ClinicalTrials.gov with the NCT02168153. The initial version sent to ClinicalTrials.gov was received on 12 June 2014.

## Abbreviations

ACT 2: Assessment of Chiropractic Treatment, part 2
AE: Adverse event
CMT: Chiropractic manipulative therapy
DC: Doctor of chiropractic
DSMC: Data and Safety Monitoring Committee
HVLA: High-velocity low-amplitude
IRB: Institutional Review Board
LBP: Low back pain
PCCR: Palmer Center for Chiropractic Research
PM: Project manager
PROMIS: Patient Reported Outcomes Measurement Information System
RCT: Randomized controlled trial
SAE: Serious adverse event
SOF: special operation forces
STaRS: Submission Tracking and Reporting System
